# 33 million year old *Myotis* (Chiroptera, Vespertilionidae) and the rapid global radiation of modern bats

**DOI:** 10.1371/journal.pone.0172621

**Published:** 2017-03-08

**Authors:** Gregg F. Gunnell, Richard Smith, Thierry Smith

**Affiliations:** 1 Division of Fossil Primates, Duke University Lemur Center, Durham, North Carolina, United States of America; 2 Directorate Earth & History of Life, Royal Belgian Institute of Natural Sciences, Brussels, Belgium; Università degli Studi di Napoli Federico II, ITALY

## Abstract

The bat genus *Myotis* is represented by 120+ living species and 40+ extinct species and is found on every continent except Antarctica. The time of divergence of *Myotis* has been contentious as has the time and place of origin of its encompassing group the Vespertilionidae, the most diverse (450+ species) and widely distributed extant bat family. Fossil *Myotis* species are common, especially in Europe, beginning in the Miocene but earlier records are poor. Recent study of new specimens from the Belgian early Oligocene locality of Boutersem reveals the presence of a relatively large vespertilionid. Morphological comparison and phylogenetic analysis confirms that the new, large form can be confidently assigned to the genus *Myotis*, making this record the earliest known for that taxon and extending the temporal range of this extant genus to over 33 million years. This suggests that previously published molecular divergence dates for crown myotines (*Myotis*) are too young by at least 7 million years. Additionally, examination of first fossil appearance data of 1,011 extant placental mammal genera indicates that only 13 first occurred in the middle to late Paleogene (48 to 33 million years ago) and of these, six represent bats, including *Myotis*. Paleogene members of both major suborders of Chiroptera (Yangochiroptera and Yinpterochiroptera) include extant genera indicating early establishment of successful and long-term adaptive strategies as bats underwent an explosive radiation near the beginning of the Early Eocene Climatic Optimum in the Old World. A second bat adaptive radiation in the New World began coincident with the Mid-Miocene Climatic Optimum.

## Introduction

Bats make up over one fifth of all living mammal species [1]. They occupy nearly every corner of the Earth and exploit a wide variety of habitats and climatic zones. Remarkably, the basic topology of the bat tree of life was established very early in their evolutionary history as they underwent a nearly instantaneous adaptive radiation during the Eocene, exploiting a previously under-utilized yet virtually limitless food resource, that of night flying insects.

Here we demonstrate the late Paleogene occurrence of the well-known living bat genus *Myotis* and document the first occurrences of extant bat and other mammalian taxa in the fossil record. We show that the presence of extant genera of major bat clades was established very early suggesting that the adaptive roles filled by these taxa were also in place very early in their diversification, roles that have been maintained to the present day. The vespertilionid bat genus *Myotis* is virtually ubiquitous with over 120 known extant species distributed around the Earth and found in nearly every geographic province except the poles and some oceanic islands [1]. In general, *Myotis* is viewed as a relatively unspecialized taxon that retains a primitive dentition [2] and, like most vespertilionids, *Myotis* lacks exaggerated morphological specializations (greatly enlarged cochlea) associated with advanced echolocating abilities [3].

Traditionally three or four subgenera of *Myotis* have been recognized based on ecologically associated morphological features that appeared to differentiate between *M*. (*Myotis*), *M*. *(Selysius*), *M*. (*Leuconoe*), and occasionally *M*. (*Cistugo*) and *M*. *(Pizon*yx) as well [4–6]. However, molecular phylogenetic analyses have repeatedly failed to support these morphological groupings [7–12] instead finding upwards of ten separate *Myotis* clades including a New World clade consisting of three subclades and an Old World clade consisting of a distinct Ethiopian clade and, at least, eight Eurasian clades [12]. Ecological groupings similar to those used to initially cluster *Myotis* species into subgenera appear in parallel within these clades [13]. Of these, only the New World and Ethiopian clades appear to be geographically circumscribed with the other clades often including taxa that, together, are broadly distributed across Eurasia.

There is an extensive fossil record of *Myotis* known predominantly from the late Oligocene through Holocene in Europe [14–21] with lesser occurrences known from the Plio-Pleistocene of Africa, the late Miocene through Pleistocene in North America, and the Pleistocene and Holocene of China, Japan and Madagascar [22–35]. In the following work a new species of *Myotis* is described from the earliest Oligocene. Following this an examination of early fossil occurrences of bat species assigned to extant genera is presented in the context of a developing scenario of separate bat adaptive radiations centered in Old and New Worlds.

**Fig 1:**
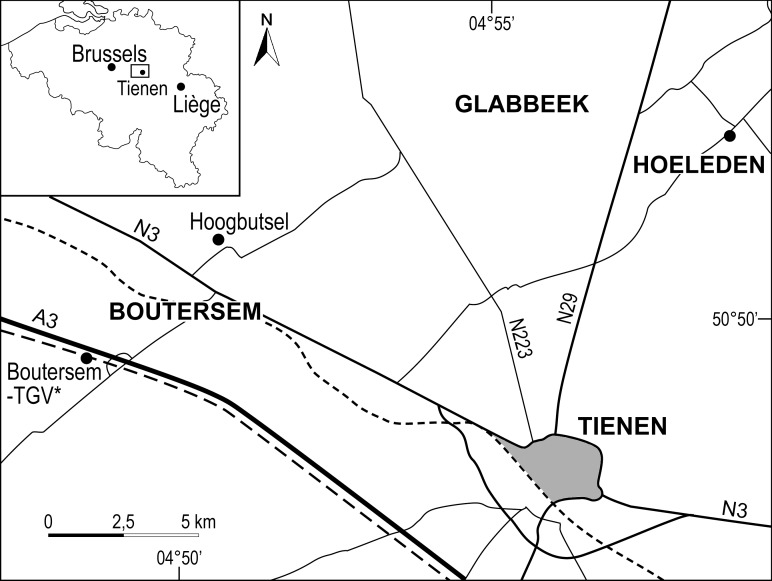
Map showing the geographic positions of the Belgian localities of Hoogbutsel, Hoeleden, and Boutersem-TGV that yielded the early myotine *Myotis belgicus* sp. nov. and the plecotine *Quinetia misonnei*.

The new *Myotis* species described here comes from the Boutersem locality in central Belgium which, along with associated localities at Hoogbutsel and Hoeleden (Fig 1), has been known since the early 1950’s and has produced a fairly extensive vertebrate faunal assemblage [36–42]. The Boutersem Sand Member belongs to the Borgloon Formation and is stratigraphically positioned just above the marine St. Huibrechts-Hern Formation located at the base of the Rupelian (earliest Oligocene), dated at 33.9 Ma [43–44]. Boutersem and its associated localities are included in European reference level MP 21 and are each approximately 33.5 million years old.

## Material and methods

### Material collected

One of us (RS) screen washed 6+ tons of matrix from the Boutersem Sand Member of the Borgloon Formation (Fig 1). No permits were required for the described study. Collection of specimens complied with all relevant local regulations and no endangered or protected species were disturbed or harmed in any way. Screen residues were then sorted under a binocular microscope and teeth and bones were extracted, mounted on pins where appropriate, identified and assigned catalog numbers. In all over 2000 vertebrate specimens were found including the 50 bat specimens described herein. The types and figured specimens described here are stored at the Royal Belgian Institute of Natural Sciences, Brussels, Belgium (RBINS = IRSNB).

### Nomenclatural acts

The electronic edition of this article conforms to the requirements of the amended International Code of Zoological Nomenclature, and hence the new names contained herein are available under that Code from the electronic edition of this article. This published work and the nomenclatural acts it contains have been registered in ZooBank, the online registration system for the ICZN. The ZooBank LSIDs (Life Science Identifiers) can be resolved and the associated information viewed through any standard web browser by appending the LSID to the prefix "http://zoobank.org/". The LSID for this publication is urn:lsid:zoobank.org:pub:A1E97058-ED37-46BB-95A5-55A7DFDD67A1. The electronic edition of this work was published in a journal with an ISSN, and has been archived and is available from the following digital repositories: PubMed Central, LOCKSS.

Standard anatomical comparisons were made with extant myotines and other vespertilionids in the collections of the RBINS and with appropriate fossil specimens of similar ages and from other circum-Tethys localities based on primary taxonomic literature (see S1 Table for a list of comparative specimens examined). Tooth terminology follows [2]. Measurements were taken either from scaled SEM images or by use of a binocular dissecting microscope fitted with a measuring reticule. Tooth measurements of fossils are presented in Tables 1 and 2.

**Table 1:** Measurements (in mm) of lower teeth of *Quinetia misonnei* and *Myotis belgicus* (L = length, W = width, H = height).

Catalog #	Genus	Species	c1 L	c1 W	c1 H	p4 L	p4 W	m1 L	m1 W	m2 L	m2 W	m3 L	m3 W
**280**	*Quinetia*	*misonnei*						1.2	0.8				
**326**	*Quinetia*	*misonnei*						1.1	0.8				
**327**	*Quinetia*	*misonnei*						1.2	0.8				
**387**	*Quinetia*	*misonnei*										1.2	0.6
**M2181**	*Quinetia*	*misonnei*				0.6	0.6						
**404**	*Quinetia*	*misonnei*						1.2	0.8	1.1	0.8		
**406**	*Quinetia*	*misonnei*								1.2	0.7		
**432**	*Quinetia*	*misonnei*								1.1	0.7		
**519**	*Quinetia*	*misonnei*								1.1	0.9	1.1	0.7
**559**	*Quinetia*	*misonnei*						1.2	0.8				
**591**	*Quinetia*	*misonnei*										1.1	0.6
**632**	*Quinetia*	*misonnei*						1.2	0.8				
**142**	*Myotis*	*belgicus*								1.5	0.8		
**150**	*Myotis*	*belgicus*								1.5	0.9		
**220**	*Myotis*	*belgicus*				1.1	0.8						
**244**	*Myotis*	*belgicus*				1.1	0.8						
**279**	*Myotis*	*belgicus*								1.5	0.8		
**325**	*Myotis*	*belgicus*										1.3	0.7
**332**	*Myotis*	*belgicus*				1	0.7						
**334**	*Myotis*	*belgicus*								1.4	1		
**M2172**	*Myotis*	*belgicus*						1.7	0.9	1.5	1	1.4	0.8
**M2173**	*Myotis*	*belgicus*				1.1	0.8						
**363**	*Myotis*	*belgicus*								1.5	1	1.4	0.9
**364**	*Myotis*	*belgicus*										1.3	0.8
**593**	*Myotis*	*belgicus*	1.1	0.9	1.5								
**612**	*Myotis*	*belgicus*				1	0.8						
**630**	*Myotis*	*belgicus*								1.5	0.9		

**Table 2:** Measurements (in mm) of upper teeth of *Quinetia misonnei* and *Myotis belgicus* (* = estimate, abbreviations as in Table 1).

Catalog #	Genus	Species	C1 L	C1 W	C1 H	P4 L	P4 W	M1 L	M1 W	M2 L	M2 W	M3 L	M3 W
**M2182**	*Quinetia*	*misonnei*								1.3	1.5		
**263**	*Quinetia*	*misonnei*						1.1	1.6				
**697**	*Quinetia*	*misonnei*								1.1	1.5		
**M2183**	*Quinetia*	*misonnei*								1.2	1.5		
**702**	*Quinetia*	*misonnei*								1.2	1.5		
**131**	*Myotis*	*belgicus*						1.6	2.3*				
**M2178**	*Myotis*	*belgicus*				1.1	1.2						
**M2179**	*Myotis*	*belgicus*						1.7	2.1				
**M2177**	*Myotis*	*belgicus*	1.1	0.9	2								
**568**	*Myotis*	*belgicus*								1.5	2		
**580**	*Myotis*	*belgicus*								1.5	2		
**592**	*Myotis*	*belgicus*	1.2	1.1	1.9								
**616**	*Myotis*	*belgicus*								1.5	2		
**M2180**	*Myotis*	*belgicus*										0.9	1.9
**Unnumbered**	*Myotis*	*belgicus*	1.2	1	1.6								

## Results

### Systematic paleontology

Class Mammalia Linnaeus, 1785

Order Chiroptera Blumenbach, 1779

Family Vespertilionidae Gray, 1821

Subfamily Myotinae Tate, 1942

Genus *Myotis* Kaup, 1829

*Myotis belgicus* sp. nov. urn:lsid:zoobank.org:act:65A2D5F3-7655-4F02-8007-F6B4C0DA18EB

#### Holotype

**Fig 2:**
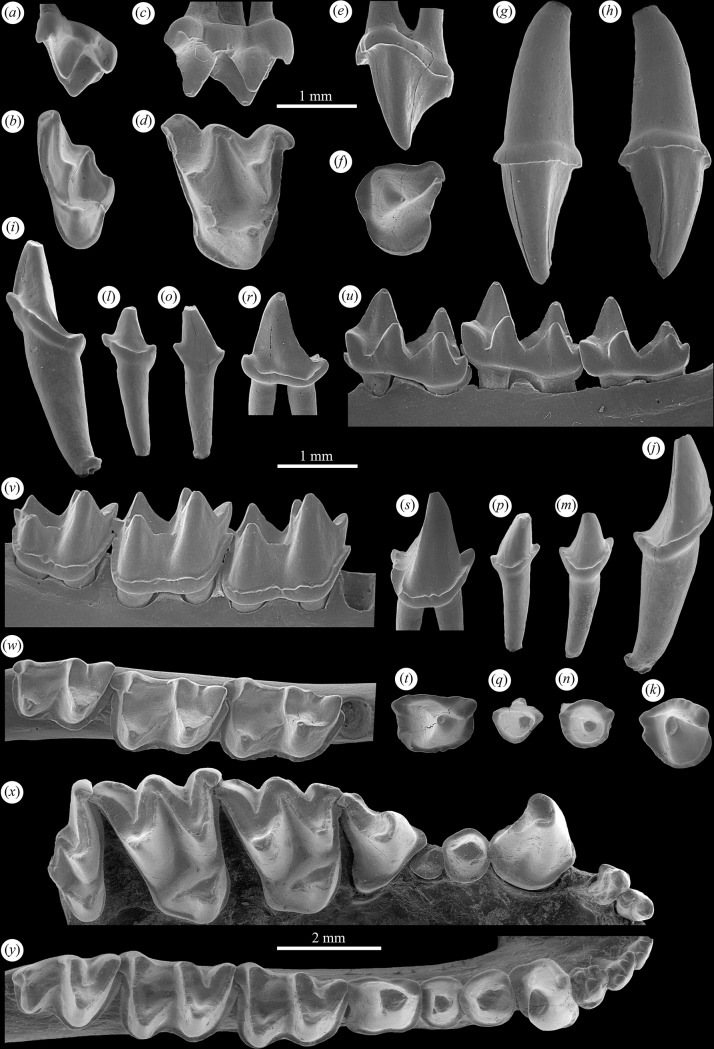
Dentition of early Oligocene myotine *Myotis belgicus* n. sp. from Boutersem, Belgium. (*a-b*) left M3, IRSNB M 2180 in labial and occlusal views; (*c-d*) right M1, IRSNB M 2179 in labial and occlusal views; (*e-f*) left P4, IRSNB M 2178 in labial and occlusal views; (*g-h*) left C1, IRSNB M 2177 in labial and lingual views; (*i-k*) right c1, IRSNB M 2176 in lingual, labial and occlusal views; (*l-n*) right p2, IRSNB M 2175 in lingual, labial and occlusal views; (*o-q*) left p3, IRSNB M 2174 in lingual, labial and occlusal views; (*r-t*) right p4, IRSNB M 2173 in lingual, labial and occlusal views; (*u-w*) right dentary m1-3, IRSNB M 2172 (Holotype) in lingual, labial, and occlusal views. Extant myotine *Myotis myotis* (*x-y*) right maxillary with I1-M3 and right dentary with i1-m3, IRSNB 98-067-0003 in occlusal views.

**Fig 3:**
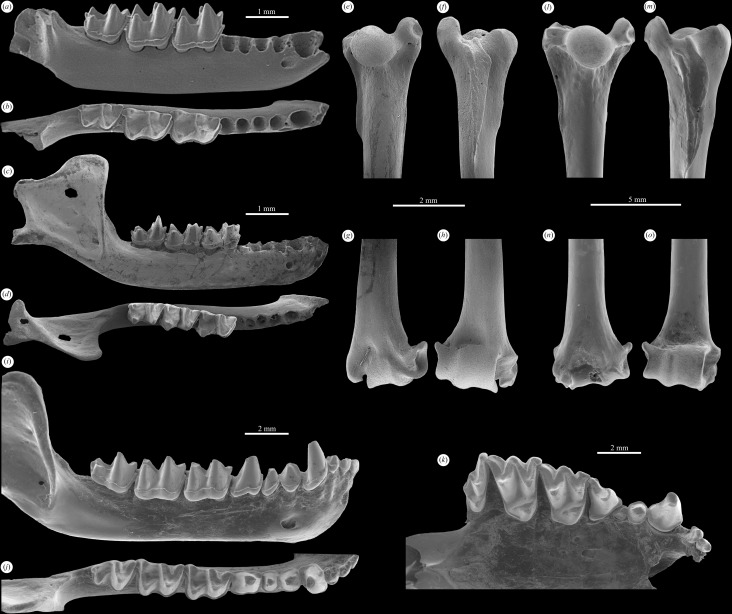
Comparison of the early Oligocene vespertilionids from Boutersem, Belgium with the extant myotine *Myotis myotis*. Early Oligocene myotine *Myotis belgicus* n. sp., IRSNB M 2172, Holotype, (*a-b*) right dentary with m1-3 and alveoli for i1-3, c, and p2-4 in labial and occlusal views. Early Oligocene vespertilionine *Quinetia misonnei*, IRSNB M 1189, holotype (*c-d*) right dentary p4-m3 (p4 now lost) and IRSNB M 2184 (*e-h*) right humerus. Extant myotine *Myotis myotis*, IRSNB 98-067-0003, (*i-j*) right dentary, (*k*) right maxillary, and (*l-o*) right humerus. Dentaries in labial and occlusal views, maxilla in occlusal view and humeri in ventral and dorsal views.

IRSNB M 2172, right dentary with m1-3 and alveoli for i1-3, c, and p2-4 (Figs 2U–2W, 3A and 3B).

#### Locality and horizon

Boutersem, Boutersem Sand Member, MP-21, Borgloon Formation, early Oligocene, Rupelian. *Myotis belgicus* is also present at Hoogbutsel, approximately 6 km northeast of Boutersem in the same formation and member.

#### Referred specimens

From Boutersem: IRSNB M 2173 (Right p4, Fig 2R–2T); IRSNB M 2174 (Left p3, Fig 2O–2Q); IRSNB M 2175 (Right p2, Fig 2L–2N); IRSNB M 2176 (Right c, Fig 2I–2Kn); IRSNB M 2177 (left C1, Fig 2G and 2H); IRSNB M 2178 (Left P4, Fig 2E and 2F); IRSNB M 2179 (Right M1, Fig 2C and 2D); IRSNB M 2180 (Left M3, Fig 2A and 2B); BOU 131 RS (Right M1); BOU 142 RS (Left m2); BOU 150 RS (Left m2); BOU 220 RS (Right p4); BOU 244 RS (Right p4); BOU 279 RS (Right m2); BOU 325 RS (Right m3); BOU 332 RS (Right p4); BOU 333 RS (Left M3 broken); BOU 334 RS (Right m1); BOU 359 RS (Right M3 broken); BOU 363 RS (Left dentary with m2-3); BOU 364 RS (Right dentary m3); BOU 405 RS (Left C); BOU 568 RS (Right M2); BOU 580 RS (Left M2); BOU 592 RS (Right C); BOU 593 RS (Left c); BOU 612 RS (Right p4); BOU 616 RS (Right M2); BOU 630 RS (Left m2).

From Hoogbutsel: IRSNB HG 1250 (Left p4); IRSNB HG 1899 (Right p2); IRSNB HG 2058 (Left C); IRSNB HG 2299 (Right p3); IRSNB HG 2365 (Left p3); IRSNB HG 2447 (Left p4); IRSNB HG 2527 (Left p4); IRSNB HG 3825 (Left c); IRSNB HG 4426 (Left p4); IRSNB HG 4591 (Left p4). See Tables 1 and 2 for tooth measurements.

#### Diagnosis

A moderately large *Myotis* species with the following combination of morphological characters: lower canine relatively low and robust with heavy lingual cingulid; p4 with distinct lingual cingulid that turns upward anteriorly to form a projecting cuspule; lower molars with relatively broad trigonid fossae and very robust hypoconulids; upper canine projecting, only slightly posteriorly curved with a continuous cingulum and distinct lingual ridge; P4 with steeply sloping postparacrista and moderate parastyle; upper molars with very weak paraloph, a short sloping postprotocrista, an anteroposteriorly broad protofossa, and two narrow but distinct ectoflexi.

#### Etymology

*Belgicus*, for Belgium where the Boutersem locality is found.

#### Description

In general, *Myotis belgicus* has about the same tooth proportions as extant *Myotis velifer*, one of the larger living species. In tooth morphology, *M*. *belgicus* is quite similar to extant *Myotis myotis* but averages 25% smaller in molar dimension than this living species (based on tooth measurements taken in the University of Michigan Museum of Zoology [UMMZ] collections).

The upper canine (Fig 2G and 2H) of *M*. *belgicus* is robust with a strong circular root that is longer in extent than the crown. The crown is circular at its base and is surrounded by a modest basal cingulid. The crown tapers to a point and has a distinctive lingual ridge that runs from base to tip and curves slightly posteriorly.

P4 (Fig 2E and 2F) has a prominent paracone and a steeply sloping paracristid that extends to a small, rounded metastylar region. There is a weak labial cingulum that expanded to form a short shelf as it wraps around the anterior aspect of the tooth where it is continuous with a flat, rounded and modestly developed lingual shelf that is not distended posteriorly.

M1 (Fig 2C and 2D) is very similar to that of *M*. *myotis* only differing in having a somewhat weaker postmetacrista, a postprotocrista that does not extend all the way to postcingulum and having a metastylar region that extends relatively farther labially. There are two distinct ectoflexi present as in *M*. *myotis*, a broader and deeper one anterior to the mesostyle and a narrower and shallower one posterior to the mesostyle.

M3 (Fig 2A and 2B) in *M*. *belgicus* differs somewhat from that of *M*. *myotis*, more resembling species such as *M*. *daubentonii*, in being relatively longer and in retaining a small metacone, a relatively long premetacrista, a distinct mesostyle, and in having a more extensive protofossa.

The lower canine (Fig 2I–2K) of *M*. *belgicus* has a tall crown with a convex anterior surface and a flattened posterior surface. It has a complete cingulid that angles towards the tip on the labial side and broadens both posteriorly and lingually, all typical *Myotis* characteristics. The posterior cingulid is notched as in some species of extant *Myotis* (e.g. *M*. *daubentonii*).

The second and third lower premolars (Fig 2L–2Q) are single-rooted teeth with a single, centered, tapering cusp dominating the crown. As in all *Myotis* species, p3 is slightly smaller than p2. Both teeth are encircled by continuous and moderately heavy cingulids.

The lower fourth premolar (Fig 2R–2T) is double-rooted and has a protoconid that is as tall as that of the molars and nearly as tall as the tip of the canine. It has a faint yet obvious preprotocristid that extends to the cingulid lingually to join a protruded cingular surface that extends towards the tip anteriorly. The labial and lingual cingulids extend posteriorly to join in a broad posterior cingular shelf with the lingual cingulid being slightly broader than the labial one. There is postprotocristid that extends down the posterolingual surface of the protoconid to join a cingulid that is distended slightly at the posterolingual border of the tooth.

The lower molars (Fig 2U–2W and Fig 3A and 3B) are typical of *Myotis* species in being myotodont with trigonids slightly taller than talonids and all major cusps present and acutely pointed. The m1 trigonid fovea is broader and more open than that of m2-3, m1 and m2 are of nearly equal size while m3 is somewhat smaller. All three molars have distinct hypoconulids (slightly smaller on m3) and strong labial cingulids that wrap around the anterior base of the teeth almost to the lingual border and extend around the posterior base of the crowns to terminate at the hypoconulid. There are no lingual cingulids developed. The cristid obliqua joins the postvallid just labial of center (m1) or nearly centrally (m2-3) and all three teeth have relatively deep talonid basins and well developed entocristids that wall off the lingual side of the talonids.

#### Comparative analysis

In addition to the phylogenetic analysis (see below), the Boutersem specimens can be assigned to *Myotis* rather than to any other vespertilionid based on the combination of the following features: 3.1.3.3 dental formula, the presence of a single-rooted p3 that is somewhat smaller than p2, myotodont lower molars that have relatively deep talonid basins, well developed entocristids and lacking lingual cingulids, a relatively high crowned lower canine with well-developed mesial and distolingual shelves, a projecting upper canine with a distinct lingual ridge, a circular cross-section and complete but not especially robust cingulum, M1 and M2 lacking both paraconules and metalophs, protofossa of M1 and M2 open posteriorly, and M3 being relatively short.

The Boutersem *Myotis* specimens represent the earliest known record of this extant genus. Only some isolated potential myotine teeth from Le Batut (MP 19) in France are older but these teeth differ from *Myotis* in having upper molars with a paraloph and a protofossa closed posteriorly, both features more typical of enigmatic “*Leuconoe*”. Myotodont species such as “*L*”. *salodorensis* from Oensingen (MP 25) in Switzerland and “*L*”. *lavocati* from Le Garouillas (MP 25–28) in France, both share features of upper teeth that distinguish them from *Myotis*, particularly in the presence of a distinct paraconule lacking in *Myotis* [21]. Younger still are three *Myotis* species from Herrlingen 8–9 (MP 29) in Germany [45]. Compared to the Boutersem *Myotis*, *M*. *minor* is much smaller with a relatively smaller, shorter and more delicate p4, *M*. *intermedius* is somewhat smaller in molar dimensions but with a substantially smaller and shorter p4, while *M*. *major* has larger m1-2, similar sized m3, smaller p4, more robust M1 and a more constricted P4 lingual shelf.

Based on its presence in Boutersem, the origin of *Myotis* must be at least as old as the early Oligocene. Slightly older *Khonsunycteris aegyptiacus* [46] from the Fayum in Egypt (34 mya) differs from *Myotis belgicus* (and all other *Myotis* species) in having p2 larger relative to p3, p2 relatively long with a distended labial surface and with a distinct preprotocristid, in having a double-rooted p3, and in having lower molars with more crestiform paraconids. Nonetheless, *Khonsunycteris* may well represent the earliest known myotine [2].

In a recent paper examining molecular relationships among approximately ¾ of the global diversity of *Myotis* species (~90 out of ~120 recognized species), Ruedi et al. [12] present evidence that crown-group *Myotis* diverged from a common ancestry with other vespertilionids (specifically a *Kerivoula-Murina* clade) at approximately 26 million years ago. Further, crown myotines are demonstrated to have diverged from enigmatic “*Myotis*” *latirostris* by approximately 21 mya. These authors suggest that because “*M*.” *latirostris*, *M*. *siligorensis alticraniatus* (subsumed into *M*. *siligorensis* by Simmons [1]) and an unnamed *Myotis* species from China all possess nyctalodont or sub-nyctalodont molars that myotodonty is not a diagnostic character of the genus *Myotis* despite the fact that virtually all of the other 117+ extant *Myotis* species and all of the 40+ fossil species of *Myotis* possess this dental characteristic to the exclusion of most other vespertilionids.

Ruedi et al. [12] also cite *Submyotodon* [21] as an example of a myotine taxon that has both sub-nyctalodont and sub-myotodont molars together in the same jaw. As it turns out, these sorts of occurrences are not entirely uncommon–Gunnell et al. [47] noted the presence of myotodont and submyotodont molars together in the myzopodid genus *Phasmatonycteris* and similar occurrences are known in some molossids [48–49] and in some archaic bats [50] where the disposition of the hypoconulid is often variable. The archaic bat *Stehlinia*, well represented from late Eocene and Oligocene Quercy deposits in France, typically is nyctalodont but some specimens of *S*. *quercyi* and *S*. *gracilis mutans* have sub-myotodont molars [51]. These examples suggest that many combinations of postcristid and hypoconulid are possible within bat species and that within a large and widespread radiation such as that of *Myotis*, some species should be expected to have developed molars that differ somewhat from the ancestral myotodont condition. However, clearly these are exceptions to an otherwise apomorphic condition shared by virtually all myotines, suggesting that the few outliers are not especially phylogenetically relevant.

Additionally, it is also important to keep in mind that it is not only the possession of myotodont molars that defines the genus *Myotis* morphologically–species of the genus also possess in combination with myotodonty the features cited above (tall lower canine with distinct distolingual cingulids, 3.1.3.3 dental formula, p3 smaller than p2 (a derived condition compared to archaic bats[50]), single-rooted p3 (derived compared to *Khonsunycteris* which has a double-rooted p3 [46]), tall, dagger-like upper canines with distinct lingual ridges (all derived compared to archaic bats [50]), P4 simple with rounded labial shelf, and upper molars lacking paraconules and metalophs (both derived compared to archaic bats [50]) and a distally open protofossa.

Ruedi et al. [12] cite the existence of *Cistugo* as another taxon sharing these same dental features with *Myotis* therefore making the assignment of *Khonsunycteris* and now *Myotis belgicus* to Myotinae less probable given the molecularly derived basal position of *Cistugo* relative to other vespertilionids [11]. However, a close inspection of the dentition of *Myotis belgicus* reveals many features in which it differs from *Cistugo* and more closely resembles *Myotis* including having: a lower canine with heavier lingual cingulid and lacking the distinctive lingual ridge that extends nearly to tip of canine in *Cistugo*; p4 with lingual cingulum turned towards tip of protoconid and forming a small cingular cuspule as in *Myotis* and unlike *Cistugo* where the cingulid is straight and flat; p4 anteroposteriorly more extensive and relatively shorter as in *Myotis*; upper molars with relatively deeper ectoflexi and a metastylar shelf that extends buccally beyond the meso- and parastyles; upper molars lacking a distinct paraloph as in *Myotis* (*Cistugo* has a distinct paraloph, a condition that more resembles *Quinetia* (see below) and “*Leuconoe*”); upper canines with less distinct cingulum and possessing a distinct lingual ridge that is absent in *Cistugo*; P4 relatively shorter with a rounded lingual shelf that is not extended distally; p2 relatively larger relative to p3 and less reduced relative to p4 as in *Myotis* and unlike in *Cistugo* where p2 and p3 are more similar in size, both small; both p2-3 with relatively higher protoconids like in *Myotis* not like *Cistugo* where these cusps are lower and equal in height.

Ruedi et al. [12] note that the divergence date for *Myotis* they predicted based on their analysis (at most 26 mya), while older than previous molecular estimates [8–10], is still nearly 7 million years younger than those suggested by paleontological evidence. Ruedi et al. [12] use only two paleontological calibrations to provide temporal constraints in their analysis—the hypothesized split of *Myotis daubentonii* and *Myotis bechsteinii* dated at between 5 and 11.6 Ma and a *Myotis* clade divergence in the late Oligocene or early Miocene (estimated by Ruedi et al. to be between 20 and 31 Ma). We suspect that by using firmer minimum dates for the first appearance of true *Myotis* (at 33.5 Ma) to provide temporal constraints, the differences in morphological and molecular divergence times for the genus would likely shrink to insignificance. Amador et al. [52] estimated a divergence time between the myotine and vespertilionine clades of 35.94 Ma, which would fit well with a first appearance of *Myotis* at 33.5 Ma.

Subfamily Vespertilioninae Gray, 1821

Tribe Plecotini Gray, 1866

*Quinetia misonnei* (Quinet, 1965)

#### Holotype

**Fig 4:**
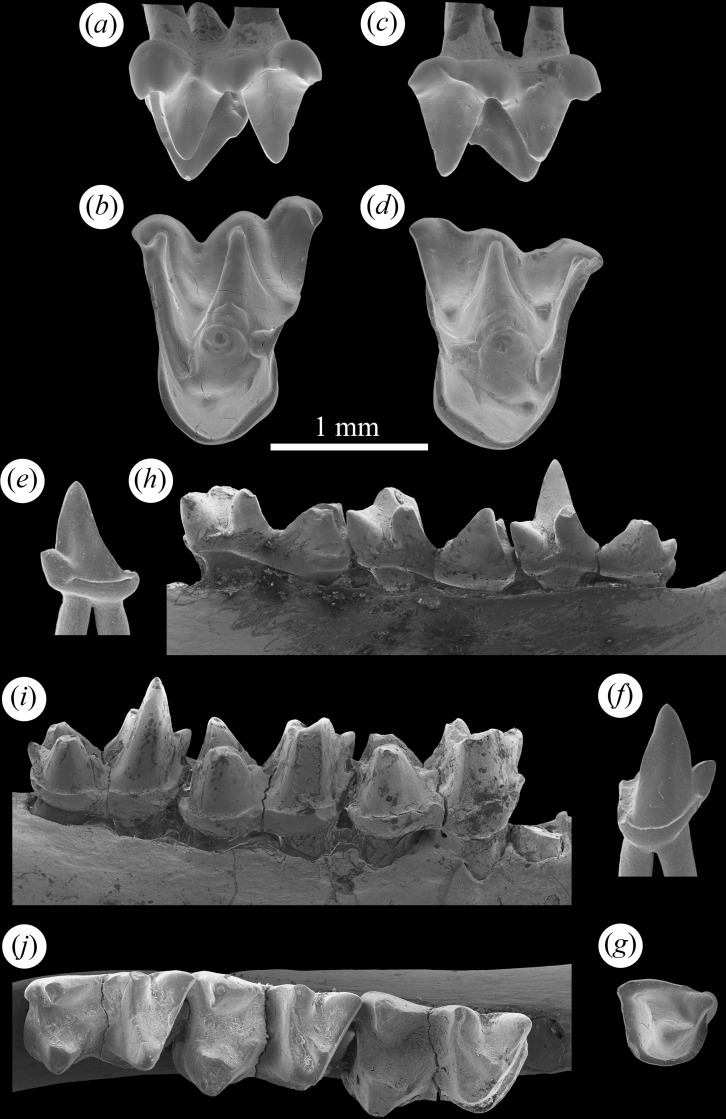
Dentition of early Oligocene vespertilionine *Quinetia misonnei* from Boutersem, Belgium. IRSNB M 2183 (*a-b*) left M2, IRSNB M 2182 (*c-d*) right M1, IRSNB M 2181 (*e-g*) p4, and IRSNB M 1189 (holotype) (*h-j*) right dentary m1-3. Upper molars in labial and occlusal views, dentary and p4 in labial, occlusal and lingual views.

IRSNB M 1189, right dentary p4-m3 (p4 now lost) (Figs 3C, 3D and 4H–4J).

#### Paratype

IRSNB M 1190, Left dentary m1-2

#### Locality and horizon

Hoogbutsel, Boutersem Sand Member, MP-21, Borgloon Formation, early Oligocene, Rupelian. *Q*. *misonnei* is also present at Boutersem, approximately 6 km southwest of Hoogbutsel in the same formation and member.

#### Referred specimens

From Hoogbutsel: IRSNB M1191 –Reg. 4200 (Edentulous dentary); IRSNB M1192 –Reg. 4201 (Left dentary with m1-2); IRSNB HG 541 (Left m1 or m2); IRSNB HG 1466 (Left C); IRSNB HG 3171 (Right c). From Boutersem: IRSNB M 2181 (Right p4, Fig 4E–4G); IRSNB M 2182 (Right M1, Fig 4C and 4D); IRSNB M 2183 (Left M2, Fig 4A and 4B); IRSNB M 2184 (Right complete humerus, Fig 3E–3H); BOU 263 RS (Right M1); BOU 280 RS (Right m1); BOU 326 RS (Right m2); BOU 327 RS (Left m2); BOU 359 RS (R M3 broken); BOU 387 RS (Right m3); BOU 404 RS (Right dentary with m1-2); BOU 406 RS (Right m2); BOU 432 RS (Right m2); BOU 519 RS (Right dentary with m2-3); BOU 538 RS (Right m3 broken); BOU 559 RS (Left m1); BOU 591 RS (Right dentary with m2-3, m2 broken, includes all anterior alveoli and ascending ramus); BOU 632 RS (Right m1); BOU 697 RS (Left M2); BOU 702 RS (Left M2); BOU 817 RS (Right p4). See Tables 1 and 2 for tooth measurements.

#### Description

*Quinetia misonnei* is represented by upper molars, a lower p4 and lower molars, a complete humerus and complete dentaries that include alveoli of all lower teeth.

The alveoli preserved in the dentary (Fig 3C and 3D) confirm the presence of the primitive bat lower dental formula of 3.1.3.3. Judging by the alveoli the canine was robust and p2 and p3 were single-rooted and nearly identical in size. The horizontal ramus is slender with a mental foramen presence below and just anterior to p2. The ascending ramus is relatively tall and straight (not leaning anteriorly), taller than is typical for extant plecotins like *Plecotus* and *Barbastella*. It has a rounded coronoid process and an articular condyle situated well above the tooth row. The angular process is broken posteriorly but appears as though it would have been extensive as in living plecotins. The mandibular fossa is relatively large and less restricted than in *Barbastella*.

The upper molars of *Quinetia* (Fig 4A–4D) resemble those of *Plecotus* more than *Barbastella* in being noticeably wider than long, with M1 being somewhat less so than M2. Both molars have two ectoflexi with those on M2 being more sharply defined and deeper. Both molars also have distinct paralophs and present, though less distinct, metalophs. As in *Plecotus*, M2 has an extended metastylar region that reaches labially beyond the para- and mesostyles. M2 has distinct hook-like para- and metastyles while only the parastyle of M1 is weakly curved. Both upper molars have moderate lingual cingula and neither shows any development of a hypocone or hypocone shelf.

The p4 of *Quinetia* (now lost from the holotype but figured previously [18, 53] is double-rooted and relatively small and short (Fig 4E–4G) as in *Plecotus*. It has a prominent protoconid and a distinct, low paraconid connected to the protoconid by a well-developed paracristid. An equally well-developed postprotocristid extends from the tip of the protoconid to the posterolingual corner of the tooth where it ends at the cingulid. There is a cingulid that nearly encircles, ending at the paraconid on both the anterolabial and anterolingual sides. The cingulid is widest posteriorly.

The lower molars of *Quinetia* are nyctalodont and have noticeably higher trigonids compared to talonids (Fig 4H–4J) like those found in most plecotins. Like *Barbastella* m3 is only somewhat reduced compared to m1-2. Unlike *Barbastella* and *Plecotus*, *Quinetia* has more closed molar trigonids with narrower trigonid fovea. The talonid of m3 in *Quinetia* is as wide as the trigonid, not narrower as in *Plecotus* and *Barbastella*. All three molars in *Quinetia* have relatively weak labial cingulids and lack any sign of lingual cingulids except a small ridge at the base of trigonid notch.

The complete humerus from Boutersem (Fig 3E–3H) is assigned to *Quinetia* based on size. The molars of *Quinetia* are very close in size to living *Myotis nigricans* which has an average humerus length (based on three specimens from the University of Michigan Museum of Zoology [UMMZ] collections) of 20.46 mm as compared to 20.0 mm for the Boutersem bat. Based on molar size, *M*. *belgicus* should have a humerus close in size to that of extant *Myotis velifer* which has an average humerus length (based on eight UMMZ specimens) of 26.06 mm. Based on these comparisons the humerus from Boutersem is more likely to be that of *Quinetia* rather than *M*. *belgicus*.

The humerus is very similar to those of extant vespertilionids (Fig 3L–3O). The trochiter (greater tuberosity) is robust and extends proximally well beyond the humeral head. The head is rounded and only slightly wider than tall. The lesser tuberosity extends to the level of the head and is rounded and robust as well. The deltopectoral crest is broad proximally, tapers distally and extends about 1/5 of the way down the shaft.

The distal end of the humerus has a trochlea only slightly more proximodistally extensive than the capitulum and continuous with it (not separated by a capitular groove). The capitulum and trochlea are aligned with the center of the shaft as is typical of vespertilionids, not offset from the shaft as in many other bats. The lateral capitular tail is narrower than the capitulum (proximodistally) and flairs laterally. The epitrochlea is not offset medially and does not extend distally beyond the surface of the trochlea. As in most vespertilionids, the distal end of the humerus is relatively narrow mediolaterally.

#### Comparative analysis

*Quinetia misonnei* was first described as a species of *Myotis* by Quinet [53]. Horáček [18] noted that *M*. *misonnei* had nyctalodont molars with shallow talonid basins and no entocristid development, in contrast to *Myotis*. He proposed the genus *Quinetia* to replace *Myotis* for this species. He also noted that these molar characteristics along with the presence of a slender and pointed p4 are quite similar to the plecotin *Barbastella*. Horáček [18] also indicated that *Q*. *misonnei* molars had well-developed lingual cingulids, a rare feature in most vespertilionids except plecotins. However, Horáček never had the opportunity to examine the type and referred specimens of *Quinetia* first-hand. We now know, based on close inspection of the type and the additional specimens from Boutersem, that *Quinetia* molars do not have lingual cingulids and, as noted by Horáček [18], *Quinetia* retains a p3 that, judging by the alveolus, was probably similar in size to or only slightly smaller than p2, both in contrast to *Barbastella*.

However, extant *Plecotus* does retain a small p3, a moderately sized p2, and has relatively short and tall p4 with a prominent anterolingual cingular cuspule, features which are also true of *Quinetia*. *Quinetia* differs from *Plecotus* in having M1-2 with para- and metalophs, a higher coronoid process of the dentary, and m1-2 with anteroposteriorly shorter (more closed) trigonids. In addition our phylogenetic analysis (see below) finds that *Quinetia misonnei* is consistently linked as the sister taxon of extant *Plecotus austriacus* supporting Horáček’s [18] notion that *Quinetia* was a probable plecotine vespertilionid.

In general, *Quinetia* appears closer to plecotins than to other vespertilionids. It clearly is more primitive in some features than extant plecotins including having distinct para- and metalophs on upper molars and a distinct paraconid on p4 but this may not be surprising given its probable position at the base of that clade. Importantly, the presence of plecotin vespertilionines at Boutersem and Hoogbutsel at 33.5 mya, implies that vespertilionines and myotines had diverged by that time (and that vespertilionines had diversified within the subfamily), adding support to the notion that the larger bats from these Belgian localities are myotines and almost certainly represent true *Myotis* as suggested here.

### Phylogenetic analysis

In order to further test the phylogenetic affinities of the bats from Boutersem, we conducted a phylogenetic analysis of a dental character matrix. The morphological data set upon which our analysis was based included 280 dental characters and 27 taxa. The matrix was built using Morphobank Version 3.0a [54] and is available for download as a TNT or NEXUS file (S1 and S2 Files). Besides *Myotis belgicus* and *Quinetia misonnei*, five other fossil taxa were scored including the archaic bat *Onychonycteris finneyi* (Onychonycteridae), the myzopodids *Phasmatonycteris butleri* and *P*. *phiomensis*, the mystacinid *Mystacina miocenalis*, and the basal myotine vespertilionid *Khonsunycteris aegypticus*. Extant taxa represent a variety of Old World Yangochiroptera including Noctilionoidea (Mystacinidae and Myzopodidae [55] although the latter family may belong in the Emballonuroidea instead [52]) and Vespertilionoidea (Miniopteridae, Cistugidae, and Vespertilionidae; see S2 Table for a list of included taxa and their current taxonomic placements). Character states were scored using original specimens, or Micro-Ct images of teeth or in some case by examining high quality casts housed at the Duke Lemur Center, Division of Fossil Primates.

All trees were rooted utilizing *Onychonycteris finneyi* as the most basal outgroup. The matrix was analyzed in TNT version 1.5 [56]. The search strategy followed that of Spaulding and Flynn [57] utilizing the ‘New Technology search’ option, selecting the sectorial search, ratchet and tree fusing search methods, all with default parameters. Under these settings, replications were run until the minimum length tree was found in 1000 separate replicates. The generated trees were then analyzed under typical search options (using TBR) to fully explore the discovered tree islands. Bremer support indices were determined using TNT and were calculated for 10 supplementary steps. Bootstrap values were calculated using TNT (1000 bootstrap replicates. Results were examined with Winclada 1.00.08 using Strict Consensus and Majority Rule trees.

**Fig 5:**
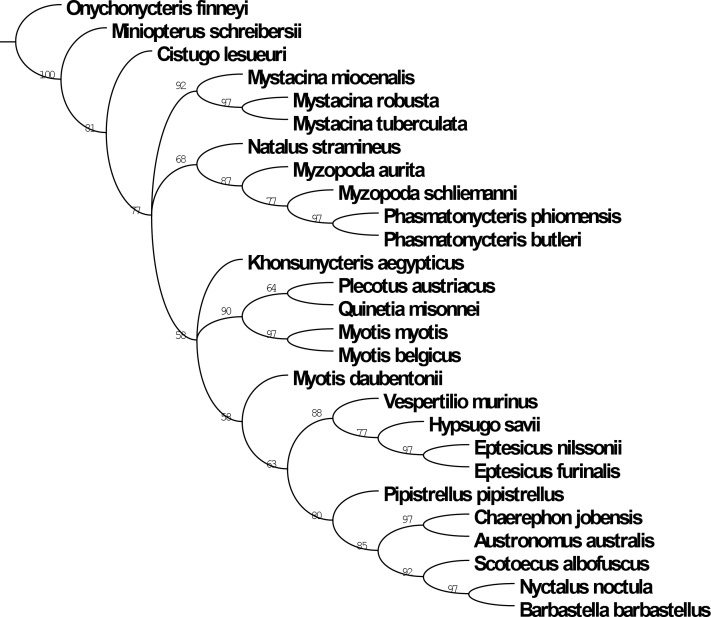
50% majority rule consensus tree of 708 steps, CI = 0.38, RI = 0.51.

The phylogenetic analysis yielded 241 equally parsimonious trees, with a tree length of 705 steps, and CI of 0.38 and RI of 0.52. The strict consensus tree is 1110 steps long with a CI = 0.24 and RI = 0.07. In the strict consensus, 24 nodes are collapsed. The majority rule consensus (Fig 5) is 708 steps long. Its CI and RI equal 0.38 and 0.51, respectively. The only value of Bremer support that TNT found is situated at the very base of the tree, between *Onychonycteris finneyi* (i.e., the most basal outgroup) and the other taxa (the value is greater than 10); this node has a Bootstrap value of 100. Two internal nodes also have Bootstrap values of 69 (for the *Austronomus-Chaerephon* clade) and 60 (for the *Myotis belgicus*-*Myotis myotis* clade)

In general the majority rule tree based on dental evidence conforms to results found based on other molecular and morphological analyses [52, 55] with some caveats (Fig 5). The separation of *Cistugo* and *Miniopterus* from Vespertilionidae into distinct families [11, 58] is supported by our analysis and we recover monophyletic Mystacinidae, Natalidae and Myzopodidae. Also *Khonsunycteris* appears as a basal vespertilionoid as has been previously suggested [2].

However, within other vespertilionoids relationships become more problematic, likely due to rampant dental homoplasy. *Myotis* is found to be paraphyletic with *M*. *daubentonii* more closely related to vespertilionines rather than other species of *Myotis*. The recognized tribes of vespertilionines are not well supported and the two molossids (*Chaerephon* and *Austronomus*) are nested within Pipistrellini along with *Barbastella* (a plecotin) and *Scotoecus* (a nycticein). These results are perhaps not surprising given the overall very similar morphology of most vespertilionoid dentitions.

The importance of this analysis for the purposes of this paper lies in the consistent linkage of *Myotis belgicus* to *Myotis myotis* to the exclusion of all other taxa. This is compelling support for including the new Boutersem species in the genus *Myotis*. The analysis also serves to confirm the likelihood that *Quinetia* is closely related to living plecotine vespertilionids and should be included in that subfamily as Horáček [18] had previously suggested.

#### Summary

The evidence presented above favors an appearance of the modern genus *Myotis* at about 33.5 mya in Europe. As noted, this date is at odds with divergence dates obtained using molecular phylogenetic reconstructions [8–12]. However, the discrepancies between morphological and molecular divergence times have begun to converge as molecular dates have gotten older [8–12]. The morphological and molecular dates for the divergence of *Myotis* are now about 7–10 million years apart but it appears that, as more evidence is accumulated, this difference is slowly decreasing.

Ruedi et al. [12] favor a geographic origin of *Myotis* in eastern Asia, either from their Eastern Palearctic or Oriental bioregions (see their Fig 3). Interestingly, these regions contain what would have been the northern shoreline of eastern Tethys during the Oligocene so perhaps, even the biogeographic region of origin supported by fossils and molecules is not so far apart either.

The fossil evidence favors an origination of basal myotines in North Africa in the later Eocene [2] followed shortly thereafter by the appearance of *Myotis* in the early Oligocene of Europe at Boutersem and Hoogbutsel. Additionally, the occurrence of *Quinetia*, a basal plecotin vespertilionine, at Boutersem provides corroborating evidence that the vespertilionid subfamilies Vespertilioninae and Myotinae had already diverged by 33.5 mya making the early occurrence of *Myotis* not especially surprising. Corroborating support of this hypothesis may be found in the presence of a bat from Prémontré in France [59] dated to 50 mya and potentially representing the earliest member of Vespertilionidae. Fossil evidence is now converging on a minimum divergence time of the family Vespertilionidae at ~ 50 mya and the divergence of Myotinae and Vespertilioninae by ~35–40 mya.

Nonetheless, it is true that *Myotis* species are very primitive bats (at least viewed in the light of what is now understood about chiropteran evolutionary trajectories [50–51]) and finding shared apomorphies between the fossil species from Belgium and recent species is very difficult (a common problem in paleontology). Despite this, based on the known evidence, it is not possible to exclude the Boutersem taxon from *Myotis* nor is it possible to identify any other extant vespertilionid that these specimens more closely resemble.

## Bat adaptive radiation

The presence of *Myotis* at 33.5 Ma in Belgium not only opens up questions about the phyletic and geographic origins of Myotinae but, in conjunction with other early occurrences of species representing extant bat genera (see below), also suggests that the early radiation of modern bats was fundamentally different from other mammalian orders.

The earliest Eocene (55.8 Ma) was a time of dramatic change in global mammalian communities as archaic Paleocene assemblages were replaced by a much more cosmopolitan and more modern communities consisting of early ancestors of many modern orders [60–61]. It has been well established based on molecular evidence [55, 62–63] that archaic bats must have partaken in this great rearrangement of communities [64] in conjunction with the Paleocene-Eocene Thermal Maximum (PETM) but to date no fossil bats have been found from deposits documenting the PETM. Therefore, it appears that the radiation of modern bats post-dates the PETM [55] and was more coincident with the onset and duration of the Early Eocene Climatic Optimum (52–50 Ma [59]).

**Fig 6:**
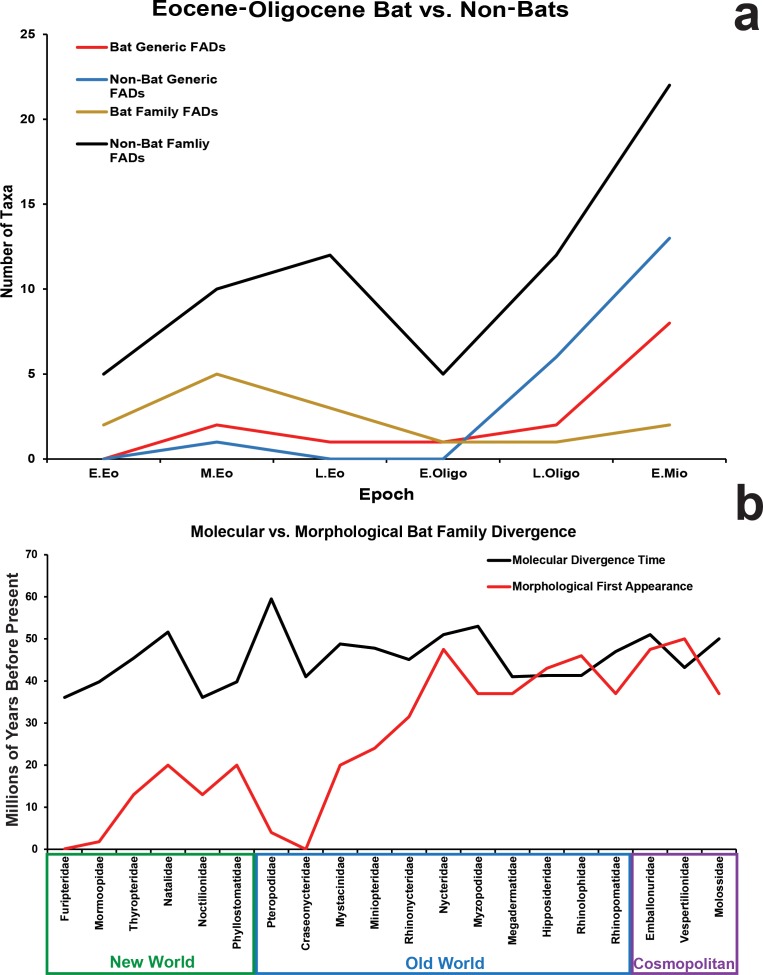
Bat global first appearance. (*a*) First appearance in the global fossil record of extant bat vs. other placental mammal families and genera from the Early Eocene through the Early Miocene. Compilation includes 15 families (75% of all extant families) and 14 genera of bats and 56 families (64% of all extant families) and 20 genera of other placentals (modified from McKenna and Bell [67], see S3 File). (*b*) Estimated molecular divergence times (black line) versus global fossil first appearances (red line) of extant bat families (molecular dates based on Teeling et al. [55] with modifications from Amador et al. [52], fossil first appearance data modified from S3 File). Families in the green box are exclusively New World in distribution today, families in the blue box are exclusively Old World, and the three families in the purple box are cosmopolitan but have Old World origins.

As fossil evidence from the Eocene has slowly been accumulating it has begun to tell a similar tale to that of molecular evidence. Archaic bats begin to appear in the early Eocene fossil records of both the Old and New Worlds [50] but as yet there are no fossil bats present in earliest Eocene faunal assemblages [50]). Modern crown-group bat families begin to appear in the later part of the early Eocene (Fig 6A) [51, 65–66].

By the middle Eocene a fundamental difference between bats and other placentals begins to become apparent. Modern bat genera begin to appear throughout the middle and late Eocene into the early Oligocene. Virtually no other mammalian group shows such early occurrences of species representing modern genera (except for a single enigmatic record of the genus *Tarsius* from the middle Eocene of China) with the earliest appearances of other living placental genera not occurring until the late Oligocene (Fig 6A).

A closer examination of which extant bat genera begin to appear early in the record reveals the presence of *Hipposideros* in the middle Eocene [51, 66, 68–69], *Rhinolophus* and *Tadarida* in the late Eocene [67–68, 70], *Myotis* in the early Oligocene (this paper) and *Megaderma* and *Mormopterus* in the late Oligocene [45, 71–72]. The presence in the Old World of two of the four major clades of echolocating bats (Rhinolophoidea and Vespertilionoidea) demonstrates that modern family level diversity has already begun to be established in the Paleogene with rhinolophoid families Hipposideridae, Megadermatidae and Rhinolophidae and vespertilionoid families Vespertilionidae and Molossidae being represented by species belonging to extant genera by that time.

While it is not possible to be absolutely certain that fossil *Hipposideros* species were filling the same adaptive roles as modern *Hipposideros* species, given the extremely similar morphology shared by each (as far as is known) it seems that it is logical to assume that they were. Today *Hipposideros* and *Rhinolophus* possess a sophisticated echolocation system (high duty cycle, constant frequency) that allows them to exploit cluttered and complex habitats often very near to the ground [73–74]. A similar style of habitat exploitation can be hypothesized for early fossil representatives of these taxa. Evidence from a fossil hipposiderid, *Tanzanycteris*, from the middle Eocene of Africa indicates, based on the presence of greatly enlarged cochlea, that this bat was already utilizing a similar echolocation system to modern hipposiderids [75].

If a similar rationale can be applied to other early appearing species of crown-group bat genera then the following can be noted (based on summaries from Nowak [73]): fossil *Tadarida* and *Mormopterus* species were probably rapid, relatively high flyers that hunted in open areas and may have lived in large colonies (especially *Tadarida*); fossil *Megaderma* species likely exploited habitats near the ground and may have preyed on small vertebrates as well as insects and roosted in small groups; fossil *Myotis* may have occupied a wide variety of habitats as living species do, were probably fairly fast and moderately high flyers that exploited areas over ponds and water courses in search of flying insects. *Myotis* typically roosts in caves today but may also roost in trees and rock hollows and depending on the season, may roost in rather large groups.

Fig 6B compares predicted molecular divergence times of bat families with fossil first appearances (FADs) and highlights an important geographical component of the bat adaptive radiation. Virtually all of the families where molecular divergences and morphological first appearances are nearly congruent are found in the Old World or among cosmopolitan groups that were first established in the Old World. Those families that have widely differing times of divergence and first appearances are almost exclusively New World taxa, taxa with extremely under-represented fossil records (Pteropodidae, Miniopteridae) or taxa of low extant diversity (Mystacinidae, Craseonycteridae, Nycteridae, Myzopodidae).

The probable reasons for the lack of congruence between molecular divergence times and morphological first appearances for New World bat clades are two-fold–the lack of a decent post-Mesozoic fossil record prior to the Late Miocene in South and Central America and the potential later arrival of ancestral noctilionoids into the New World [47]. If ancestral noctilionoids did not reach the New World until the latest Eocene or early Oligocene this could help to explain a second explosive adaptive radiation of bats (best typified by Phyllostomidae) in the New World in the Miocene [76].

**Fig 7:**
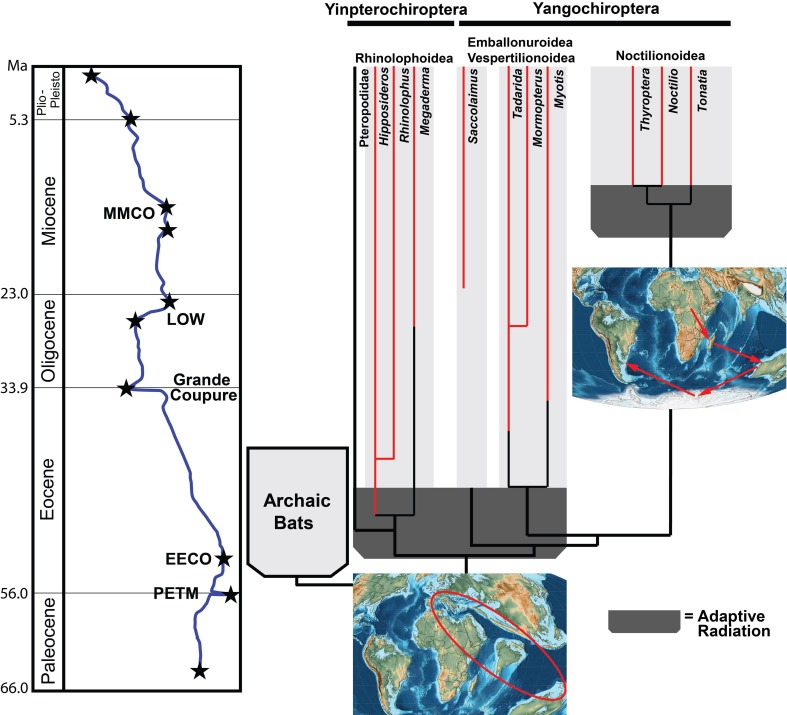
Proposed trajectory of bat evolutionary history. Light-filled tapered rectangle represents the archaic bat radiation beginning near the Paleocene-Eocene boundary and ending at the beginning of the Oligocene, coincident with drop in global temperatures. Dark-filled tapered rectangles represent modern bat adaptive radiations, one in the Old World coincident with the Early Eocene Climatic Optimum and a second one in the New World coincident with the Mid-Miocene Climatic Optimum. Paleotemperature curve in blue with black stars indicating PETM (Paleocene-Eocene Thermal Maximum), EECO (Early Eocene Climatic Optimum), the Grande Coupure, LOW (Late Oligocene Warming Event), and the MMCO (Mid-Miocene Climatic Optimum. Red lines indicate range of selected modern genera of bats, red oval on Old World map indicates probable area where both archaic and modern bats first arose, and red arrows on circum-southern oceanic area indicates the probable origin and route taken by ancestral noctilionoids to reach the New World [47].

It is becoming increasingly clearer that the geographic origins of crown-group Chiroptera are centered in the circum-Tethys region [50, 66] (Fig 7). The earliest known records of confirmed bats come from Europe, India, North Africa and Australia from localities all dating to around 53–54 mya [50, 65, 77–78] but all of these represent archaic bat groups that have no clear phylogenetic connections with modern taxa. Modern families followed almost immediately by some modern genera of bats began to appear in the Old World in the late early to middle Eocene of Europe and North Africa [66]. The appearance of extant generic level taxa representing differentiated clades within Rhinolophoidea and Vespertilionoidea so early in the Paleogene is evidence of rapid diversification and suggests that the adaptive roles played by species within these genera were established very early and seemingly continue to the present day.

## Conclusions

Bat evolutionary history as now understood (Fig 7) can be best visualized as consisting of the following phases: 1) an Old World early archaic phase centered around the ancient Tethys Sea wherein early bats develop many defining characteristics (flight, echolocation, roosting behavior) either from an ancestry in the New World (North American *Onychonycteris* and *Icaronycteris* [3,50]) or from *in situ* origination near Tethys region [50]; 2) an Eocene rapid adaptive radiation of crown-group bat taxa in the Old World coincident with the onset of the Early Eocene Climatic Optimum wherein bats undergo rapid diversification into night flying, insect predating forms while developing modifications of flight and echolocating abilities in order to fully exploit an aerial hawking life-style [59, 79–80]–it is during this radiation that within-community niches apparently were established and were begun to be occupied by species of extant genera in the Old World; and 3) a second rapid diversification of noctilionoids, coincident with the Mid-Miocene Climatic Optimum, after their ancestors arrived in the New World in the latest Eocene or early Oligocene [81–82]. This New World radiation produced a remarkable taxonomic diversity as well as broad morphological disparity across an array of dietary specializations (fruit eaters, nectar feeders, insect specialists of many kinds, blood consuming vampires, and animal and invertebrate consuming specialists) among noctilionoids bats, especially Phyllostomidae [1, 76, 83–84]. In both the Old World and the New World after the initial establishment of extant bat families in the Eocene and Miocene respectively, these communities rapidly diversified and apparently remained relatively stable throughout the course of the rest of the Cenozoic.

## Supporting information

S1 File Taxon-character matrix utilized in phylogenetic analysis in TNT format. (TNT)

S2 File Taxon-character matrix utilized in phylogenetic analysis in NEXUS format. (NEX)

S3 File First appearance data of placental mammals in the fossil record. (XLSX)

S1 Table List of comparative specimens used during this study. (DOCX)

S2 Table Classification of species employed in phylogenetic analysis. (DOCX)
