# Supplementary material for: 33 million year old Myotis (Chiroptera, Vespertilionidae) and the rapid global radiation of modern bats
Source: PLoS One. 2017 Mar 8;12(3):e0172621. doi: 10.1371/journal.pone.0172621 (PMC5342209; doi:10.1371/journal.pone.0172621)
Supplement: S1 Table — (DOCX) [file pone.0172621.s004.docx]

**S1 Table. List of extant bat comparative specimens used during this study.**

**American Museum of Natural History Specimens**

Miniopteridae

AMNH 218977, *Miniopterus schreibersi*, female, Australia

Vespertilionidae

AMNH 197234, *Chalinolobus gouldii*, female, Australia

AMNH 74902, *Corynorhinus rafinesque*, USA

AMNH 235371, *Eptesicus furinalis*, Brazil

AMNH 86640, *Hypsugo anehietai*, male, Angola

AMNH 87244, *Laephotis angolensis*, male, Angola

AMNH 130229, *Lasionycteris noctivagans*, Jamaica

AMNH 140425, *Lasionycteris noctivagans*, USA

AMNH 134921, *Lasiurus blossevillii*, female, Brazil

AMNH 144815, *Lasiurus borealis*, USA

AMNH 214126, *Lasiurus cinerus*, female, USA

AMNH 253712, *Lasiurus intermedius*, male, USA

AMNH 139871, *Lasiurus seminolus*, Bermuda

AMNH 234207, *Murina suilla*, female, Malaysia

AMNH 48937, *Neoromicia nanus*, male, DRC

AMNH 184356, *Neoromicia rendalli*, female, Sudan

AMNH 257398, *Nycticeinops schlieffeni*, South Africa

AMNH 104835, *Nycticeius sanborni*, Papua

AMNH 212071, *Otonycteris hemprichii*, male, Baluchistan

AMNH 81089, *Pipistrellus hesperidus fuscatus*, female, Ethiopia

AMNH 185329, *Pipistrellus nanulus*, male, West Africa

AMNH 182924, *Rhogeessa tumida*, male, Trinidad

AMNH 241054, *Scotoecus albofuscus*, female, Cameroon

AMNH 237386, *Scotoecus albofuscus*, female, Kenya

AMNH 184960, *Scotoecus hindei*, female, Sudan

AMNH 257434, *Scotophilus dinganii*, female, South Africa

AMNH 162705, *Vespadelus caurinus*, female, Australia

**Field Museum of Natural History Specimens**

Miniopteridae

FMNH 28765, *Miniopterus africanus*, Ethiopia

FMNH 169681, *Miniopterus manavi*, Madagascar

**Forschungsinstitut Senckenberg Specimens**

Miniopteridae

SMF 30.178, *Miniopterus australis*, Luzon

SMF 83.522, *Miniopterus gleni*, West Madagascar

SMF 85.768 (Male)/SMF 84.728 (female), *Miniopterus magnater* Laos (male), Vietnam (female)

SMF 89.116, *Miniopterus majori*, West Madagascar

SMF 44.665, *Miniopterus medius*, Malaysia

SMF 65.199, *Miniopterus pusillus*, Thailand

Vespertilionidae

SMF 88.662, *Myotis ater*, Thailand

SMF 82.968, *Myotis bechsteinii*, Germany

SMF 49.233 (female)/SMF 38.912 (male), *Myotis blythii blythii*, Afghanistan

SMF 34.253, *Myotis blythii oxygnathus*, Spain

SMF 43.391, *Myotis blythii punicus*, Tunisia

SMF 47.776, *Myotis blythii punicus*¸ Morocco

SMF 90.872, *Myotis blythii punicus*, Libya

SMF 36.960, *Myotis capaccini*, Turkey

SMF 38.916, *Myotis emarginatus desertorum*, Afghanistan

SMF 50.429, *Myotis emarginatus emarginatus*, Corsica

SMF 59.656, *Myotis emarginatus emarginatus*, Algeria

SMF 58.751, *Myotis formosus*, Afghanistan

SMF 66.036, *Myotis hasselti*, Thailand

SMF 29.551, *Myotis jeannei*, Luzon

SMF 38.756, *Myotis longipes*, Afghanistan

SMF 78.311, *Myotis myotis*, Germany

SMF 91.348, *Myotis nipalensis transcaspicus*, Iran

SMF 69.338, *Myotis ridleyi*, Malaysia

SMF 88.683, *Myotis rosseti*, Thailand

SMF 49.594, *Myotis scotti*, Ethiopia

SMF 53.317, *Myotis siligorensis*, Thailand

**Royal Ontario Museum specimens**

Vespertilionidae

ROM 58424, *Myotis adversus*, Australia

ROM 86357, *Myotis annectans*, Sumatra, female

ROM 78744, *Myotis blythii*, Lebanon

ROM 83948, *Myotis bocagei*, Kenya, male

ROM 79256, *Myotis dasycneme*, Netherland

ROM 35678, *Myotis daubentoni*, Czech Republic, female

ROM 112102, *Myotis gomantongensis*, Malaysia, female

ROM 38005, *Myotis horsfieldi*, Malaysia, female

ROM 39660, *Myotis macrotarsus*, Philippines

ROM 40943, *Myotis muricola*, Malaysia, male

ROM 35345, *Myotis myotis*, Belgium

ROM 102816, *Myotis mystacinus*, United Kingdom, male

ROM 102812, *Myotis nattereri*, United Kingdom, male

ROM 76272, *Myotis ricketti*, Hong Kong

ROM 78625, *Myotis tricolor*, Kenya, male

ROM 91210, *Myotis weltwitschii*, Kenya, male

**University of Michigan Museum of Zoology specimens**

Molossidae

UMMZ 163620, *Chaerephon plicata*, female

Miniopteridae

UMMZ 59015, *Miniopterus schreibersi*

UMMZ 103548, *Miniopterus schreibersi*, male

UMMZ 111011, *Miniopterus schreibersi*, female

UMMZ 123520, *Miniopterus schreibersi*, male

UMMZ 123521, *Miniopterus schreibersi*, female

UMMZ 157001, *Miniopterus schreibersi*, female

Vespertilionidae

UMMZ 90482, *Antrozous pallidus*, female

UMMZ 125333, *Eptesicus furinalis*, male

UMMZ 125741, *Eptesicus furinalis*, male

UMMZ 125742, *Eptesicus furinalis*, female

UMMZ 125743, *Eptesicus furinalis*, female

UMMZ 125744, *Eptesicus furinalis*, female

UMMZ 133789, *Eptesicus furinalis*, female

UMMZ 98959, *Eptesicus fuscus*, female

UMMZ 172254, *Hesperoptenus tickelli*, female

UMMZ 111018, *Idionycteris phyllotis*, male

UMMZ 161398, *Kerivoula whiteheadi*, female

UMMZ 106057, *Lasionycteris noctivagans*, male

UMMZ 89680, *Lasiurus borealis*, female

UMMZ 97160, *Lasiurus borealis*, male

UMMZ 97161, *Lasiurus borealis*, male

UMMZ 99793, *Lasiurus borealis*, male

UMMZ 99794, *Lasiurus borealis*, female

UMMZ 99975, *Lasiurus borealis*, female

UMMZ 112546, *Murina cyclotis*, female

UMMZ 112547, *Murina cyclotis*, female

UMMZ 112548, *Murina cyclotis*, female

UMMZ 172226, *Murina cyclotis*, female

UMMZ 33093, *Myotis lucifugus*, female

UMMZ 109335, *Myotis myotis*, male

UMMZ 123523, *Myotis myotis*, female

UMMZ 156907, *Philetor brachypterus*, male

UMMZ 172292, *Pipistrellus javanicus*, female

UMMZ 112327, *Plecotus phyllotis*, male

UMMZ 115743, *Plecotus rafinesquii*, male

UMMZ 99984, *Plecotus townsendi*, female

UMMZ 157014, *Scotophilus kuhlii*, male
