# Supplementary material for: 33 million year old Myotis (Chiroptera, Vespertilionidae) and the rapid global radiation of modern bats
Source: PLoS One. 2017 Mar 8;12(3):e0172621. doi: 10.1371/journal.pone.0172621 (PMC5342209; doi:10.1371/journal.pone.0172621)
Supplement: S2 Table — (DOCX) [file pone.0172621.s005.docx]

**S2 Table. Classification of species employed in phylogenetic analysis (†= extinct taxon).**

**Archaic Chiroptera**

**†Onychonycteridae**

†*Onchyonycteris finneyi*

**Yangochiroptera**

**Noctilionoidea**

**Mystacinidae**

†*Mystacina miocenalis*

*Mystacina robusta*

*Mystacina tuberculata*

**Emballonuroidea**

**Myzopodidae**

*Myzopoda aurita*

*Myzopoda schliemanni*

†*Phasmatonycteris phiomensis*

†*Phasmatonycteris butleri*

**Vespertilionoidea**

**Vespertilionoid incertae sedis**

†*Khonsunycteris aegypticus*

**Miniopteridae**

*Miniopterus schreibersi*

**Cistugidae**

*Cistugo lesueuri*

**Natalidae**

*Natalus stramineus*

**Vespertilionidae**

**Vespertilioninae**

**Eptesicini**

*Eptesicus nilssonii*

*Eptesicus furinalis*

**Nycticeiini**

*Scotoecus albofuscus*

**Pipistrellini**

*Nyctalus noctula*

*Pipistrellus pipistrellus*

**Plecotini**

*Barbastella barbastellus*

*Plecotus austriacus*

†*Quinetia misonnei*

**Vespertilionini**

*Hypsugo savii*

*Vespertilio murinus*

**Myotinae**

*Myotis myotis*

†*Myotis belgicus*

*Myotis daubentonii*

**Molossidae**

*Chaerephon jobensis*

*Austronomus australis*
